# Anticancer Activity of Indian Stingless Bee Propolis: An *In Vitro* Study

**DOI:** 10.1155/2013/928280

**Published:** 2013-05-16

**Authors:** Milind K. Choudhari, Reihaneh Haghniaz, Jyutika M. Rajwade, Kishore M. Paknikar

**Affiliations:** Centre for Nanobioscience, Agharkar Research Institute, G. G. Agarkar Road, Pune 411004, India

## Abstract

Indian stingless bee propolis has a complex chemical nature and is reported to possess various medicinal properties. In the present study, anticancer activity of the ethanolic extract of propolis (EEP) was explored by testing the cytotoxic and apoptotic effect in four different cancer cell lines, namely, MCF-7 (human breast cancer), HT-29 (human colon adenocarcinoma), Caco-2 (human epithelial colorectal adenocarcinoma), and B16F1 (murine melanoma), at different concentrations. Cytotoxicity was evaluated by MTT assay and Trypan blue dye exclusion assay. EEP at a concentration of 250 *μ*g/mL exhibited ≥50% mortality in all cell lines tested (i.e., IC_50_ value). EEP revealed a concentration and time dependent cytotoxic effect. Apoptosis was estimated by differential staining (ethidium bromide/acridine orange) and TUNEL (deoxynucleotidyl transferase-dUTP nick end labeling) assay. Light microscopy and atomic force microscopy demonstrated morphological features of apoptosis in all the cell lines after treatment with 250 *μ*g/mL EEP for 24 h. Thus, early onset of apoptosis is the reason for anticancer activity of Indian stingless bee propolis. Further, the antioxidant potential of Indian stingless bee propolis was demonstrated to substantiate its anticancer activity.

## 1. Introduction 

Propolis is a complex resinous material that bees collect from tree exudates primarily resins of leaf bud and other botanical sources. It is mixed with beeswax to create a sealing material in their comb, smooth out the internal walls, and protect the entrance against intruders [1]. Color of propolis varies depending on its botanical source and age mostly it possess yellowish green to dark brown and aromatic odor [2]. Propolis contains predominantly phenolic compounds including flavonoids and cinnamic acid derivatives [3]. Propolis has a long history of being used in traditional medicine dating back to 300 BC [2] and has been reported to have a broad spectrum of biological activities, namely, anticancer, antioxidant, antiinflammatory, antibiotic and antifungal activities [4, 5]. Recently, propolis is marketed in various forms such as tablets, capsules, toothpaste, mouthwash preparations, face cream, ointments, lotions, and solutions [6]. Studies have already shown anticancer properties of propolis and its phenolic components by different mechanisms such as cell cycle arrest, induction of apoptosis [7], induction of mitochondrial stress [8], inhibition of cancer cell proliferation and tumor growth [9–12]. Moreover, reports suggest antioxidant properties for propolis samples using different chemical assays, such as scavenging of DPPH radical [13] and scavenging of superoxide anion [14], Chemical composition of propolis mainly varies depending upon its origin, which in turn, would result in change in the activity of propolis [15]. There have been several reports on anticancer activity of propolis from various parts of the globe. These interesting reports prompted us to study the anticancer potential of Indian stingless bee propolis. Indian stingless bee propolis has a complex composition with 24 different chemical compounds. In our previous communication, we reported potent broad spectrum antimicrobial activity of ethanolic extract of propolis (EEP) [16]. Here, we report the anticancer activity of EEP on different cancerous cells based on cytotoxcity assays, apoptogenic potential, and antioxidant activity. 

## 2. Material and Methods

### 2.1. Preparation of Propolis

Propolis sample was collected from a colony of Trigona bees in the village Karjat (Western Ghats), Raigad District, Maharashtra, India. EEP was prepared according to the method described by Muli et al. [16, 17]. In brief, collected propolis sample was airdried and powdered (in a mortar and pestle), and 3 g powdered sample was admixed with 70% ethanol (10 mL) and macerated for a week. The macerated ethanolic extract was filtered through Whatman # 41 filter paper and used as EEP.

### 2.2. Cells and Culture Conditions

Cell lines used in this study were human breast adenocarcinoma (MCF-7), human colon adenocarcinoma (HT-29), human epithelial colorectal adenocarcinoma (Caco-2), and murine melanoma cell lines (B16F1). Cell lines were procured from the National Center for Cell Science (NCCS), Pune, India. Cells were grown in Dulbecco's Modified Eagle's Medium (DMEM) (Invitrogen, Grand Island, USA) supplemented with 10% heat inactivated fetal bovine serum (FBS) (Invitrogen, Grand Island, USA), 100 U/mL penicillin G, and 100 *μ*g/mL streptomycin (HiMedia, Mumbai, India) maintained at 37°C in humidified air with 5% CO_2_. 

### 2.3. Cytotoxicity of EEP

Cytotoxicity of EEP in four different cell lines (B16F1, MCF-7, HT-29, and Caco-2) was determined by MTT (3-(4,5-dimethylthiazol-2-yl)-2-5 diphenyl tetrazolium bromide) assay. MTT is captured by cells and reduced intracellularly in a mitochondrion-dependent reaction to yield a formazan product. The ability of cells to reduce MTT provides an indication of their intactness and mitochondrial activity that serves as a measure of viability [18]. Cells were plated in 96-well tissue culture plate at a density of 1 × 10^4^ cells/well and incubated for 24 h at 37°C. Thereafter, the cells were exposed to different concentrations (10, 25, 50, 100, and 250 **μ**g/mL) of EEP for 24 h. Cells cultured in the absence of EEP served as control. Cytotoxicity was assessed by adding MTT (HiMedia, Mumbai, India) dissolved in medium at a concentration of 5 mg/mL (10 *μ*L of MTT/well), followed by incubation for 4 h at 37°C in a humid 5% CO_2_ atmosphere. Further, the supernatant was removed, and the insoluble formazan crystals were dissolved in 200 **μ**L dimethylsulfoxide (Sisco Research Laboratories, Pvt. Ltd., Mumbai, India). The absorbance was measured at 570 nm using microplate reader (Synergy HT, Bio-Tek Instruments Inc., USA). Assay was performed in triplicates and repeated twice. The percentage of viability was calculated using the following formula:

(1) $$\begin{array}{c} \%\;\text{Cell}\;\text{viability}=\frac{{\text{OD}}_{570}\;\text{treated}\;\text{cells}}{{\text{OD}}_{570}\;\text{control}}\times 100. \end{array}$$

Data obtained were analyzed using One-way ANOVA followed by Dunnett's multiple comparison test to identify statistically significant differences in cell viability in comparison to respective control. *P* values of <0.05 were considered statistically significant. On the basis of cytotoxicity result, the concentration of EEP showing ≥50% reduction in cell viability was then considered as the IC_50_ value.

### 2.4. Trypan Blue Dye Exclusion Assay

To assess time dependent cytotoxicity, direct counting of living and dead cells after exposure to cytotoxic concentration (as determined by MTT assay above) of EEP was carried out using trypan blue dye exclusion assay. B16F1, MCF-7, HT-29, and Caco-2 cells (5 × 10^5^) were incubated with EEP (final concentration of 250 *μ*g/mL) for different time periods, namely 1, 3, 6, 12, and 24 h. Untreated cells served as control. Cells from test and controls were harvested using trypsin phosphate versene glucose (TPVG) (HiMedia, Mumbai, India) at each mentioned time points. Resultant cell suspension was then admixed with 0.4% Trypan blue dye (HiMedia, Mumbai, India) and counted in Neubauer chamber and viable cells were estimated by the following formula [19]:

(2) $$\begin{array}{c} \text{Viable}\;\text{cells}=\frac{\text{Unstained}\;\text{cells}}{\text{Stained}\;\text{cells}+\text{Unstained}\;\text{cells}}. \end{array}$$

### 2.5. Cell Morphology

For microscopy, cells were cultivated on cover slips and treated with EEP at concentrations of 50 and 250 *μ*g/mL for 24 h at 37°C, under 5% CO_2_ atmosphere. Subsequently, cover slips were washed twice with PBS and used for imaging. Morphological and confluency changes in treated versus untreated (control) cells were observed using an inverted microscope (Nikon Eclipse, TS-100, Japan).

Topological changes were visualized in all four cell lines using atomic force microscopy (AFM) (MultiView-1000, Nanonics Imaging Ltd., Jerusalem, Israel). The AFM scanning was performed in intermittent contact mode, using glass fiber probes (resonant frequency: 35–37 KHz, Nanonics Imaging Ltd., Jerusalem, Israel). Images were processed using WSxM software (4.0 Develop 8.1, Nanotec Electronica, S.L., Spain). 

### 2.6. Analysis of Apoptosis by Acridine Orange/Ethidium Bromide Staining (AO/EB)

To observe nuclear morphology and differentiate apoptotic and necrotic cell death, cells were stained with acridine orange (AO) and ethidium bromide (EB). In brief, 1 × 10^5^ cells (all four cell lines) were cultured on glass cover slips for 24 h at 37°C. Thereafter, the cells were treated without (control) or with EEP (at the dosage determined as cytotoxic value by MTT assay, i.e., 250 *μ*g/mL) for 24 h. After trypsinisation, cells were washed twice with PBS and stained by adding sufficient mixture of AO/EB (100 *μ*g/mL in PBS) for 5 min. Cells were immediately visualized by fluorescence microscope (Nikon 80i, Japan) at 200x magnification with the excitation filter 480/30 nm. Three independent cell counts (counting a minimum of 200 total cells) were obtained on the basis of differential staining of nuclei. Acridine orange is taken up by both live and dead cells and emits green fluorescent, whereas ethidium bromide stains only dead cells which lost their membrane integrity and emits red fluorescent [20].

### 2.7. TUNEL Assay

Cells undergoing DNA fragmentation were detected using terminal deoxynucleotidyl transferase-dUTP nick end labeling (TUNEL) kit (Click-iT TUNEL Alexa Fluor Imaging Assay; Invitrogen, Grand Island, USA). Cells were seeded on cover slips in 6-well plates until 80% confluency was obtained. Cultured cells were then incubated without and with EEP (250 *μ*g/mL) for 24 h. Further, cells were fixed with 4% chilled paraformaldehyde (Sigma-Aldrich, MO, USA) in phosphate buffer saline (PBS) for 15 min and permeabilized (0.25% Triton X-100 in PBS; Sigma-Aldrich, MO, USA) for 20 min at room temperature. Subsequent steps were as per manufacturer's protocol. Finally, cover slips were washed with PBS and observed under a fluorescence microscope.

### 2.8. DPPH Radical Scavenging Assay

For determination of free radical scavenging activity of EEP, DPPH^•^ (1,1-diphenyl-2-picrylhydrazyl; Sigma-Aldrich, MO, USA) radical scavenging assay was performed. Trolox (the commercially available antioxidant; Sigma-Aldrich, MO, USA) was used as a positive control. 1 mL of DPPH reagent (75 *μ*M in methanol) and 200 *μ*L of test samples in 70% methanol were incubated at 37°C for 80 min. The reduction of the absorbance at 515 nm (UV 2450 spectrophotometer, Shimadzu) was monitored and expressed as mg of Trolox equivalent DPPH radical-scavenging activity. The experiment was performed in triplicate. IC_50_ (concentration providing 50% inhibition) value was calculated by using the dose inhibition curve in a linear range by plotting the extract concentrations versus the corresponding scavenging effect [21].

## 3. Results

### 3.1. Cytotoxicity of EEP

**Figure 1:**
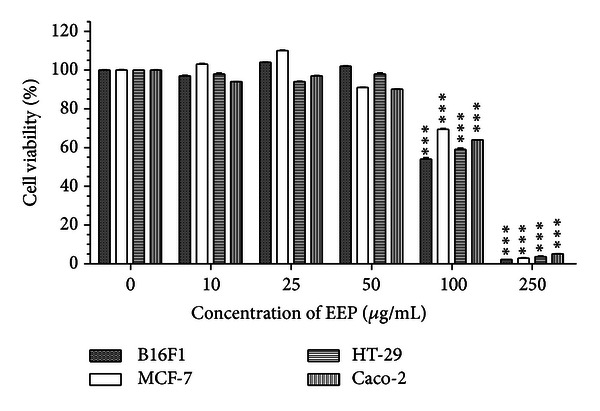
Concentration effectiveness of ethanolic extract of propolis (EEP) on cell viability of four cancer cell lines: B16F1, MCF-7, HT-29 and Caco-2. Values are represented as mean ± SEM of three replicates. ***Represents significant value (*P* < 0.01) tested with one-way ANOVA when the treated group was compared with the respective control group.

When compared with the respective control, viability of cells significantly reduced after treatment with higher concentrations of EEP for 24 h (Figure 1). IC_50_ value was found to be 250 *μ*g/mL for all the tested cell lines. EEP concentrations up to 50 *μ*g/mL were noncytotoxic for all the cell lines tested since 90% cell survival was observed. However, at 100 *μ*g/mL, a reduction in viability was obtained which was statistically significant. In summary, the results indicate concentration-dependent cytotoxic effect of EEP in all the cell lines tested.

### 3.2. Trypan Blue Dye Exclusion Assay

**Figure 2:**
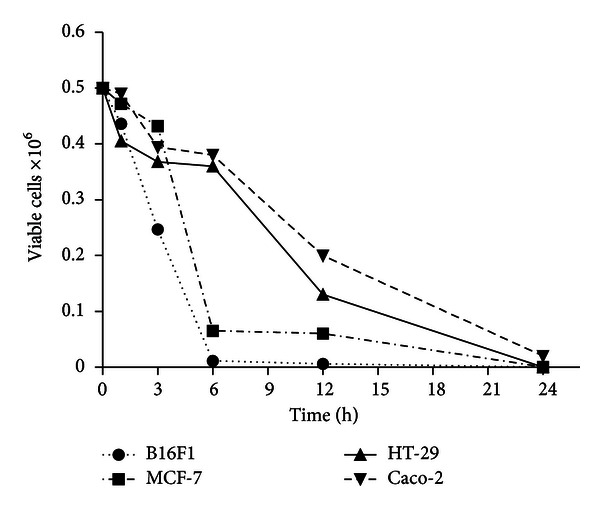
Cell death determination by trypan blue exclusion dye assay in four cell lines which showed increase in cell death in a time dependent manner.

As seen in Figure 2, in all the cell lines treated with concentration of 250 *μ*g/mL (i.e., IC_50_ value), progressive reduction in cell viability was observed over a period of 24 h. In fact, at 24 h there was a complete loss of cell viability in all the cancer cell lines. Interestingly, the cell viability data at intermediate time point of 3 and 6 h showed differences with respect to the cell lines used. At 3 h, >75% cells of MCF-7, HT-29, and Caco-2 retained viable, whereas at 6 h ≤10% viability was observed for B16F1 and MCF-7. At 12 h, cell viability in B16F1, MCF-7, HT-29, and Caco-2 cells was found to be ~1%, 12%, 26%, and 40%, respectively. Data suggest time-dependent cytotoxic effect of EEP. 

### 3.3. Cell Morphology

**Figure 3:**
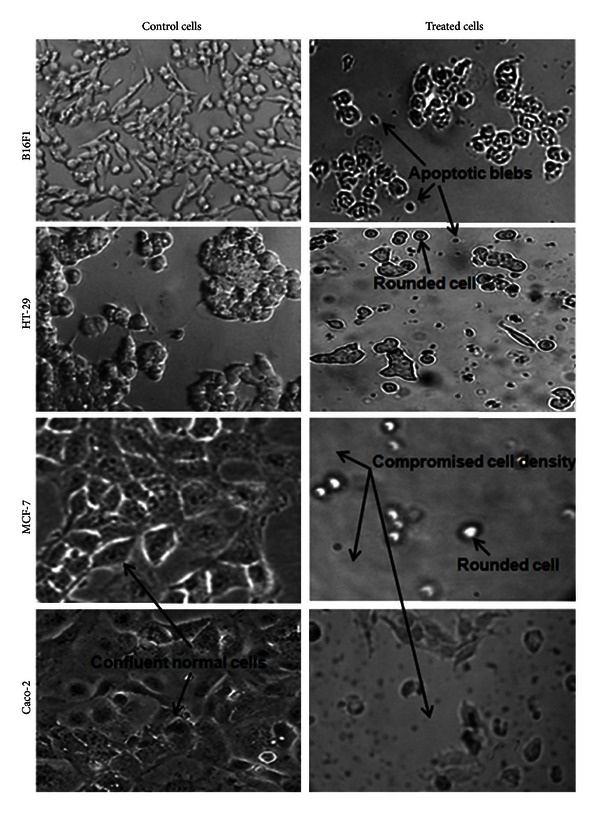
Inverted microscopy micrographs of four different cancer cell lines before and after treatment with 250 *μ*g/mL of EEP for 24 h at actual magnification of 100x. Control cells demonstrated dense cell populations with very few rounded cells. Treated cells showed increased numbers of rounded cells with compromised cell density and suspended appearance with apoptotic blebs.

**Figure 4:**
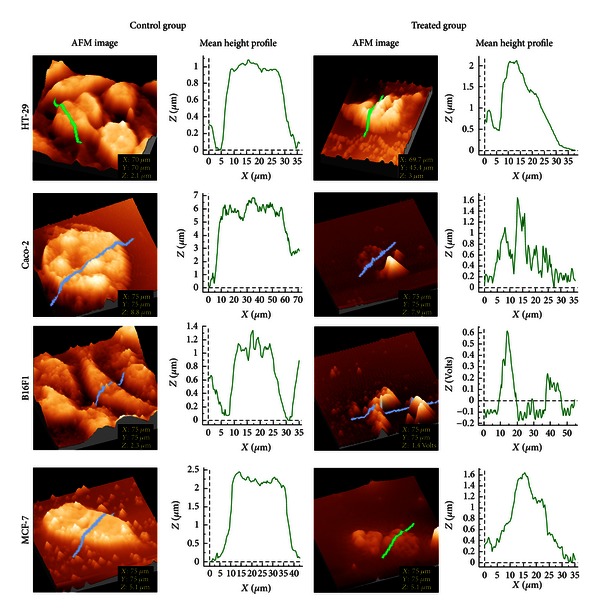
AFM 3D images for control and treated (with 250 *μ*g/mL EEP) group. Mean height profile (height versus width graph) representing topology along the selected area in each image. Change in the surface topology can be seen visually by AFM image and graphically by Mean height profile.

Optical microscopic observations on cells cultivated in the presence of EEP revealed that there were no morphological alterations at concentrations up to 50 *μ*g/mL in all the four cell lines and cell morphology matched with that of control cells. However, cells treated with higher concentrations of EEP (i.e., ≥100) revealed drastic changes in cell morphology. Cells exposed to these doses showed a loss of cell extensions, rounding up and detachment, apoptotic blebbing, and reduction in size as well as cell density as compared to untreated cells (Figure 3). Further, to observe any topological changes in the cell membrane, AFM imaging was carried out. Obtained images were converted in a 3D format, and mean height profile was represented graphically using the software (Figure 4). In case of untreated (control) cells, clearly defined boundaries were observed although cell surface appeared rough. At 50 *μ*g/mL EEP, the shape of the cells was similar to that of control. At the concentration of 100 *μ*g/mL, cell morphology was not entirely disturbed; however, the surface topology showed alterations. After exposure to 250 *μ*g/mL EEP for 24 h, the cellular morphology was completely disturbed. Surface topology was severely altered with respect to cell height and surface roughness. Moreover, the cell membrane was practically indistinguishable as it is shown in Figure 4. The mean height profile spanning a single cell is illustrated at the right side of each AFM image (Figure 4). Overall, the results obtained by AFM are in agreement with optical microscopy. Irrespective of the cell lines tested the above observations were similar.

### 3.4. Analysis of Apoptosis by AO/EB Staining

**Figure 5:**
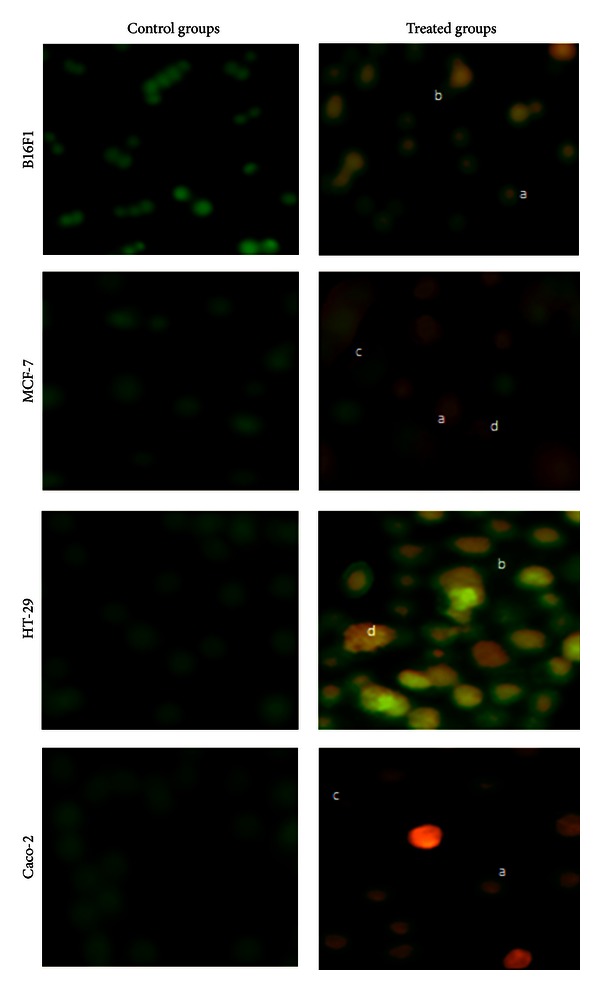
Morphological analysis of apoptosis by AO/EB staining in four different cancer cell lines at actual magnification of 200x. (a) The figure indicates cells with chromatin condensation; (b) indicates membrane blebbing; (c) indicates the presence of apoptotic bodies and (d), indicates cells with fragmented chromatin.

**Figure 6:**
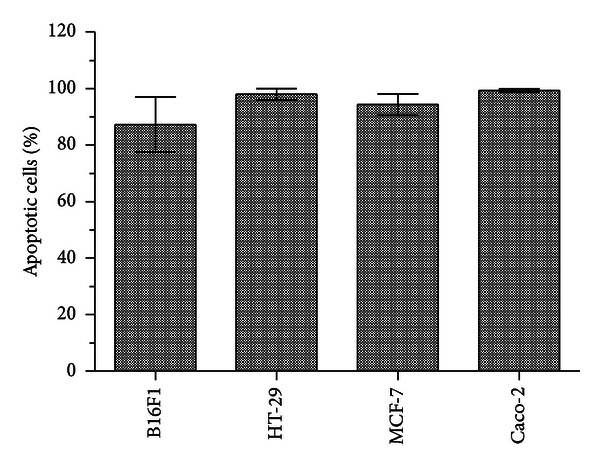
Induction of apoptosis in four cancer cell lines after 250 *μ*g/mL EEP treatment. Minimum of 200 total cells counted in three different fields after AO/EB staining.

In order to elucidate the mechanism of cell death induced by EEP in all the cancer cells, a simple method based on microscopic observations of cells stained with AO/EB was performed. AO/EB staining revealed uniform green nucleus in all cells that were not exposed to EEP (Figure 5; control cells). However, morphological features of apoptosis were observed in all the cell lines after treatment with 250 *μ*g/mL EEP for 24 h (Figure 5). Yellow staining demonstrated early apoptotic cells, whereas reddish or orange staining suggested late apoptosis which was predominantly observed in treated MCF-7 cells. Typical characteristics such as (Figure 5; arrows in treated cells) shrinkage in nucleus, alteration in shape of cells, membrane blebbing, nuclear fragmentation and chromatin condensation were visualized in the apoptotic cells of all cell lines. Necrosis (characterized by a structurally normal orange nucleus) was not observed in any of the cell lines exposed to 250 *μ*g/mL of EEP. On the basis of cell counts (Figure 6), maximum percentage of apoptotic cells was observed in case of Caco-2 and HT-29 (99.3% and 98%, resp.) with least apoptosis in B16F1 cells (87.2%). It needs to be noted that the percentage of apoptotic cells in all the respective control groups was less than 5%. 

### 3.5. TUNEL Assay

**Figure 7:**
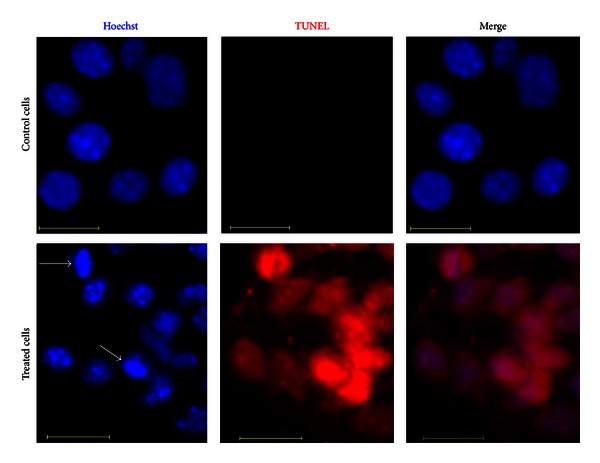
Fluorescence micrographs of HT-29 cells treated with 250 *μ*g/mL of EEP for 24 h. Viable cells did not show red fluorescence, whereas apoptotic cells emitted red fluorescence. White arrows indicate chromatin condensation due to apoptosis. Scale bar is 20 *μ*m.

As per our observations, >99% of cells treated with EEP (250 *μ*g/mL) were TUNEL positive. The TUNEL assay demonstrated cell death primarily due to DNA fragmentation in B16F1, MCF-7, Caco-2, and HT-29 cell lines after exposure to 250 *μ*g/mL EEP for 24 h. Representative image for HT-29 cells is shown in Figure 7. The nucleus of treated cells showed bright blue fluorescence as compared to the normal nuclei in the respective controls. Moreover, reduction in size of nucleus was observed in treated cells, indicating chromatin condensation and fragmentation as markers characteristic of apoptosis. 

### 3.6. DPPH Radical Scavenging Assay

The efficient concentration required for decreasing initial DPPH concentration by 50% (IC_50_) was calculated by plotting a dose response curve for EEP. The IC_50_ value for Indian stingless bee propolis was found to be 153 *μ*g/mL. DPPH radical scavenging activity was expressed as mg of Trolox equivalents per mg of the sample which was 460 ± 0.64.

## 4. Discussion 

With cancer being a fatal disease, there has been several efforts to treat cancer using various natural and synthetic materials. Due to problems such as undesirable side effects of chemotherapeutic agents, their drug resistance, complementary and alternative medicine is emerging as a possible solution. Epidemiological data support the concept that naturally occurring anti-cancer agents in the human diet are safe, non-toxic, and have long-lasting beneficial effects on human health [22, 23].

Many reports have indicated that different types of propolis extracts significantly inhibit cell growth and reduce the differentiation or proliferation of tumor cells [12, 24]. Moreover, cytotoxicity may largely vary in different samples of propolis. Szliszka et al. [12] reported 50 *μ*g/mL EEP from southern Poland, exhibited 25% cytotoxicity in prostate cancer cells. Vatansever et al. [25] showed that EEP at a concentration of 125 *μ*g/mL is cytotoxic in MCF-7 cell line and also reported differences in cytotoxic effects of seven different EEP samples collected from the same location. These observations indicate that chemical composition and pharmacological activities vary according to geographical and botanical origin of propolis. Therefore, it was interesting to find out toxicity effect of Stingless Bee Propolis of Indian origin. In the present study, we investigated the anti-cancer effects of Indian EEP on four different cancer cell lines. Our results demonstrated lower cytotoxicity effect of EEP evaluated by MTT assay (i.e., 250 *μ*g/mL) as compared to reported value [12, 25], which can be attributed to its different geographical origin. Moreover, there are reports on anticancer drug resistance cell lines such as Caco-2 cells. It has been reported that Caco-2 cells show resistance to doxorubicin, due to drug efflux mediated via P-glycoprotein (P-gp) [26]. Therefore, there is a need of introducing new anticancer drug for such kind of resistant cells. Interestingly, our finding showed that Caco-2 cells are susceptible to Indian origin EEP; however, it needs further* in vivo* evaluation.

Further, cell viability test based on trypan blue exclusion dye assay was assessed at 250 *μ*g/mL concentration (i.e., IC_50_). The results obtained suggest time-dependent cytotoxicity effect of EEP in all the cell lines tested. These data are in agreement with the study by Franchi Jr. et al. [27] where the authors reported a decline in cell viability within 24 and 48 h after treatment with two different types of propolis collected from the southeastern region in Brazil.

Our morphological observations of cells upon exposure to different concentrations of EEP, using optical microscopy and AFM corroborate with the cytotoxicity assay results. Our microscopic observations, at 100 *μ*g/mL itself showed a loss of cell extension, rounding up and detachment. However, it does not give an idea about the surface topology of cells. AFM is a tool for surface topology examination. To the best of our knowledge this is the first report of using AFM imaging to show cell morphology changes due to EEP. Here, we observed significant topological changes in cells by both the microscopic techniques, which showed changes typical of apoptosis namely, apoptotic membrane blebbing and detachment of cells. Similar results have been reported by Vatansever et al. where they have shown morphological changes in MCF-7 cells after treatment with apoptotic dose of propolis [25].

Apoptosis is an important phenomenon in chemotherapeutic agent induced killing of cancer cells. Apoptosis induction is one of the mechanisms proposed for the therapeutic effects of propolis [8, 11]. To investigate possible mechanism of cell death due to EEP treatment we performed differential staining (using AO/EB) and TUNEL assay. Our results indicated that mode of action of EEP is by inducing apoptosis, since DNA fragmentation is evidenced by TUNEL assay. Vatansever et al. have shown induction of caspases in MCF-7 cells [25]. Szliszka et al. discussed augmentation of TRAIL-induced apoptotic death in prostate cancer cells due to EEP [12]. In case of Indian stingless bee propolis, its selective influence on cells and the pathways involved in its proapoptotic activity needs to be studied further.

It is well known that propolis possesses antioxidant property which can be associated with the active principles associated with its anticancer activity [4]. Antioxidant property of propolis was studied by DPPH radical scavenging assay. In a study by Lu et al. [28], variations in antioxidative activities of EEP in terms of scavenging DPPH free radicals depending on the location and time period of collection are discussed. In their study, the potencies of free radical-scavenging activities vary from 17.90 to 108.05 *μ*g/mL. Our results indicated a potent antioxidant activity of Indian stingless bee propolis which is 153 *μ*g/mL. Variations in antioxidant property as assessed by DPPH radical scavenging activity could be attributed to differences in the geographical location and botanical origin of propolis [28, 29]. Nagai et al. [29] demonstrated the anti-oxidative activity in commercially available propolis. They postulated that flavonoids, such as quercetin, flavones, isoflavones, flavonones, anthocyanins, catechin and isocatechin may contribute to the anti-oxidative activity they observed. Also, other studies have shown antioxidant property of propolis and correlated it to its complex composition mostly consisting flavonoids, cinnamic acid derivatives and phenolic compounds [30, 31]. Presence of such compounds in Indian stingless bee propolis supports its anti-oxidant potential. 

## 5. Conclusion

In conclusion, Indian stingless bee propolis has a potent anticancer activity on cell lines tested, and it causes cell death due to induction of apoptosis. The study also draws attention to the antioxidant potential of Indian stingless bee propolis. Further *in vitro *studies are needed to investigate the mechanism of proapoptotic activity of EEP.

## List of Abbreviations

EEP: Ethanolic extract of propolis
NCCS: National Center for Cell Science
DMEM: Dulbecco's Modified Eagle's Medium
FBS: Fetal bovine serum
MTT: 3-(4,5-Dimethylthiazol-2-yl)-2-5 diphenyltetrazolium bromide
TPVG: Trypsin phosphate versene glucose
AFM: Atomic force microscopy
AO: Acridine orange
EB: Ethidium bromide
PBS: Phosphate buffer saline
TUNEL: Terminal deoxynucleotidyl transferase-dUTP nick end labeling
DPPH: 1,1-Diphenyl-2-picrylhydrazyl.
